# General Microbiota of the Soft Tick *Ornithodoros turicata* Parasitizing the Bolson Tortoise (*Gopherus flavomarginatus*) in the Mapimi Biosphere Reserve, Mexico

**DOI:** 10.3390/biology9090275

**Published:** 2020-09-05

**Authors:** Sergio I. Barraza-Guerrero, César A. Meza-Herrera, Cristina García-De la Peña, Vicente H. González-Álvarez, Felipe Vaca-Paniagua, Clara E. Díaz-Velásquez, Francisco Sánchez-Tortosa, Verónica Ávila-Rodríguez, Luis M. Valenzuela-Núñez, Juan C. Herrera-Salazar

**Affiliations:** 1 Unidad Regional Universitaria de Zonas Áridas, Universidad Autónoma Chapingo, 35230 Bermejillo, Durango, Mexico; sergiokun.barraza@gmail.com (S.I.B.-G.); cmeza2020@hotmail.com (C.A.M.-H.); 2 Facultad de Ciencias Biológicas, Universidad Juárez del Estado de Durango, 35010 Gómez Palacio, Durango, Mexico; vavilar@gmail.com (V.Á.-R.); luisvn70@hotmail.com (L.M.V.-N.); hsjc20@ujed.mx (J.C.H.-S.); 3 Facultad de Medicina Veterinaria y Zootecnia No. 2, Universidad Autónoma de Guerrero, 41940 Cuajinicuilapa, Guerrero, Mexico; homero.ppca@gmail.com; 4 Laboratorio Nacional en Salud, Diagnóstico Molecular y Efecto Ambiental en Enfermedades Crónico-Degenerativas, Facultad de Estudios Superiores Iztacala, 54090 Tlalnepantla, Estado de México, Mexico; felipe.vaca@gmail.com (F.V.-P.); cdiazvelasquez@aol.com (C.E.D.-V.); 5 Instituto Nacional de Cancerología, 14080 Ciudad de México, Mexico; 6 Unidad de Biomedicina, Facultad de Estudios Superiores Iztacala, Universidad Nacional Autónoma de México, 54090 Tlalnepantla, Estado de México, Mexico; 7 Departamento de Zoología, Universidad de Córdoba.Edificio C-1, Campus Rabanales, 14071 Cordoba, Spain; ba1satof@uco.es

**Keywords:** microbiota, soft ticks, Bolson tortoises, bacterial profile, tick-borne pathogens

## Abstract

The general bacterial microbiota of the soft tick *Ornithodoros turicata* found on Bolson tortoises (*Gopherus flavomarginatus*) were analyzed using next generation sequencing. The main aims of the study were to establish the relative abundance of bacterial taxa in the tick, and to document the presence of potentially pathogenic species for this tortoise, other animals, and humans. The study was carried-out in the Mapimi Biosphere Reserve in the northern-arid part of Mexico. Bolson tortoises (*n* = 45) were inspected for the presence of soft ticks, from which 11 tortoises (24.4%) had ticks in low loads (1–3 ticks per individual). Tick pools (five adult ticks each) were analyzed through 16S rRNA V3–V4 region amplification in a MiSeq Illumina, using EzBioCloud as a taxonomical reference. The operational taxonomic units (OTUs) revealed 28 phyla, 84 classes, 165 orders, 342 families, 1013 genera, and 1326 species. The high number of taxa registered for *O. turicata* may be the result of the variety of hosts that this tick parasitizes as they live inside *G. flavomarginatus* burrows. While the most abundant phyla were Proteobacteria, Actinobacteria, and Firmicutes, the most abundant species were two endosymbionts of ticks (*Midichloria*-like and *Coxiella*-like). Two bacteria documented as pathogenic to *Gopherus* spp. were registered (*Mycoplasma* spp. and *Pasteurella testudinis*). The bovine and ovine tick-borne pathogens *A. marginale* and *A. ovis*, respectively, were recorded, as well as the zoonotic bacteria *A. phagocytophilum,* *Coxiella burnetii*, and *Neoehrlichia* sp. Tortoises parasitized with *O. turicata* did not show evident signs of disease, which could indicate a possible ecological role as a reservoir that has yet to be demonstrated. In fact, the defense mechanisms of this tortoise against the microorganisms transmitted by ticks during their feeding process are still unknown. Future studies on soft ticks should expand our knowledge about what components of the microbiota are notable across multiple host–microbe dynamics. Likewise, studies are required to better understand the host competence of this tortoise, considered the largest terrestrial reptile in North America distributed throughout the Chihuahuan Desert since the late Pleistocene.

## 1. Introduction

Ticks are widely distributed in the world as obligate hematophagous ectoparasites of reptiles, birds, and mammals [1]. In recent years, traditional culture media practices have been replaced by high-throughput techniques such as the next generation sequencing, NGS, used to explore the complete bacterial microbiota of ticks [2]. It is known that tick microbiota is mainly composed of endosymbionts and tick-borne pathogens [3]. Endosymbionts play several beneficial roles to the host (fitness, development, reproduction, immunity, nutritional adaptation, and defense against environmental stress) [4], and tick-borne pathogens are of great concern to animal and human health around the world [3,5]. These two groups of microorganisms form a complex and dynamic ecosystem within ticks, coexisting and interacting with them in a commensal, mutualistic, or pathogenic way [3,6]. From a biological point of view, it is important to know the bacteria that compose of the tick microbiota, but in an epidemiological sense it is fundamental to develop information about the presence and abundance of tick-borne pathogens especially in countries where there is very scarce data.

Tick-borne bacteria have been largely studied in hard ticks (family Ixodidae) *Amblyomma* spp., *Dermacentor* spp., *Haemaphysalis* spp., *Hyalomma* spp., *Ixodes* spp., and *Rhipicephalus spp.*, where zoonotic bacteria *Anaplasma phagocytophilum*, *Coxiella burnetii*, *Ehrlichia chaffensis*, *Francisella tularensis*, and *Rickettsia rickettsi* have been reported [7,8,9,10]. In soft ticks (family Argasidae), most studies have focused on the genus *Ornithodoros*, which comprises of more than 60% of the family [1,11]. These ticks usually inhabit sheltered sites (i.e., burrows, caves, cracks, and nests), commonly parasitizing small mammals, birds, reptiles, or bats [12]. Particularly, *O. turicata* is distributed through the arid regions of the Southern United States and Latin America [13]. It has been reported frequently as a promiscuous feeder parasitizing wild animals such as ground squirrels, prairie dogs, snakes, and desert tortoises [14]. In Mexico, this tick has been reported as an ectoparasite of the Bolson tortoise, *Gopherus flavomarginatus* [15], an endemic and endangered reptile species restricted to the Chihuahuan desert [16,17]. *Gopherus* tortoises are considered keystone species because they dig burrows where they spend the most part of its life, providing additional shelter for other animals such as rodents, lagomorphs, birds, and reptiles [18]. However, it is in their burrows where they most likely to acquire soft ticks. Due to the ecological relevance that *G. flavomarginatus* represents for the Chihuahuan desert ecosystem, it is important to generate information on the possible pathogens with which it may have contact, in this case those transmitted by soft ticks. Since there is no previous information regarding the bacterial microbiota of *O. turicata* in Mexico, we established the relative abundance of bacterial taxa in this tick. We hypothesized that the *O. turicata* microbiota will include potentially pathogenic bacteria for the Bolson tortoises, other animals, and even humans.

## 2. Materials and Methods

### 2.1. General

All the methods and activities of this study were in strict accordance with accepted guidelines for ethical use, care, and welfare of animals in research at the international level [19]. The federal approval reference number is SEMARNAT-SGPA/DGVS/08406/16. This research has been approved by the Facultad de Ciencias Biológicas UJED ethic committee on 5 February 2017 (ethic code: 0023). The files used in this study (Supplementary files: P1.fasta, P2.fasta and P3.fasta) were deposited into the NCBI Sequence Read Archive (SRA) database (SRA Accession Number: PRJNA649587). 

### 2.2. Location and Environmental Conditions

**Figure 1:**
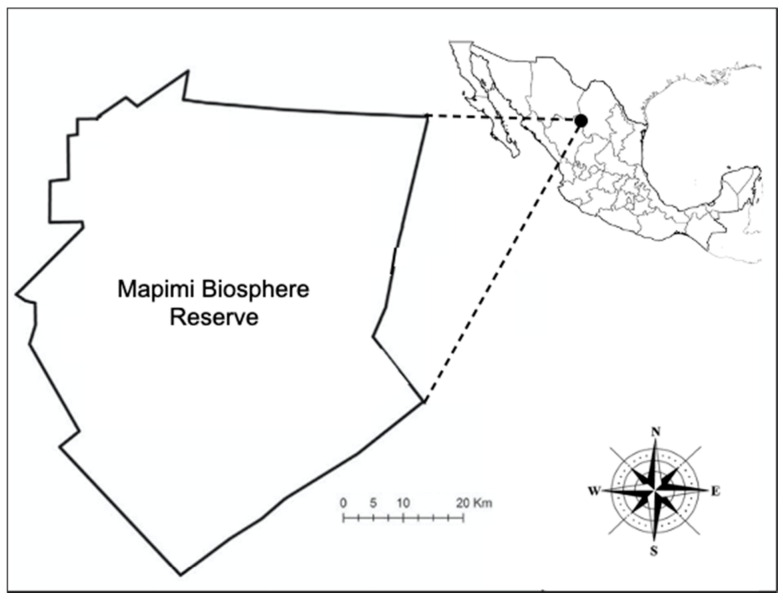
Geographic location of the Mapimi Biosphere Reserve in Northern Mexico.

The study was carried-out in the Mapimi Biosphere Reserve in Mexico, which includes part of the states of Chihuahua, Coahuila, and Durango (26°00′ and 26°10′ N, 104°10′ and 103°20′ W; Figure 1). The Bolson de Mapimi has been defined as an endoreic basin, which includes diverse small sub-basins intermixed along valleys with a mean altitude of 1150 m. This area has very arid climate [20], with an average annual temperature of 25.5 °C, and an average annual precipitation of 264 mm [21]. The predominant vegetation in the reserve is rosette and microphile scrub, as well as halophyte, and gypsophila plants [22]. Interestingly, the microregion concentrates the richest herpetofauna across the whole Chihuahuan desert, having diverse endemic species, including the Bolson tortoise [23].

### 2.3. Field Work

**Figure 2:**
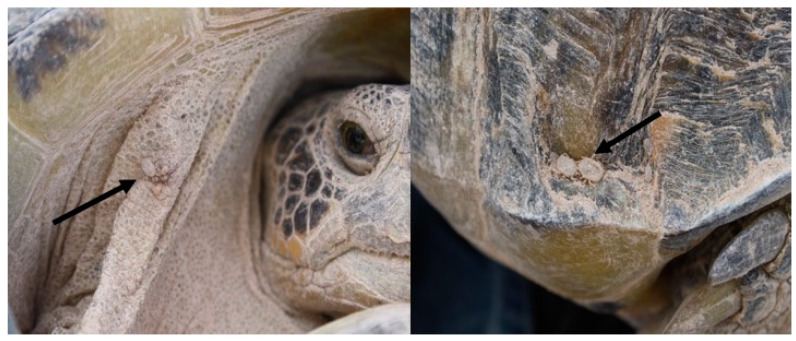
*Ornithodoros turicata* soft ticks parasitizing the Bolson tortoise (*Gopherus flavomarginatus*) in the Mapimi Biosphere Reserve, Mexico.

**Figure 3:**
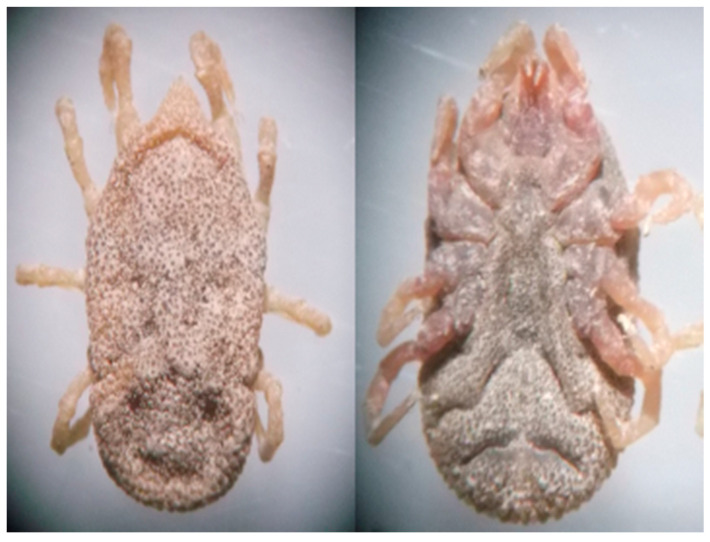
*Ornithodoros turicata* soft tick (adult) parasitizing the Bolson tortoise (*Gopherus flavomarginatus*) in the Mapimi Biosphere Reserve, Mexico. Left is the dorsal view and right is the ventral view.

From May to July 2017, Bolson tortoises were captured by hand from 900 to 1300 h and from 1700 to 2100 h. The search for ticks was carefully carried out on the carapace, neck and skin folds of all four limbs. A total of 45 individuals of *G. flavomarginatus* were captured and revised, but only 11 tortoises (24.4%) carried soft ticks on the carapace or over the skin (Figure 2 and Figure 3). Tortoises carried from one to three ticks per individual. A total of 17 adult ticks were collected and taxonomic keys were used to determine the species [24]. Each tick was placed in an individual 1.5 mL tube containing 500 uL ethanol (70%), 500 uL hydrogen peroxide (H_2_O_2_), and 200 uL of ultrapurified H_2_O; each tube was vortexed for 15 s to remove tick surface contaminants [25]. Later, three pools were formed (five ticks each) and were deposited in BashingBead™ Zymo Research™ cell lysis tubes, containing 750 μL of lysing/stabilizing solution. The tubes were processed in a cellular disruptor (TerraLyzer™) for 20 s. The two extra ticks were deposited in the Entomological collection of the Facultad de Ciencias Biológicas, Universidad Juárez del Estado de Durango, México, for reference purposes.

### 2.4. DNA Extraction, Visualization, and Quantification

DNA was extracted from the pools using the Xpedition™ Tissue/insect DNA MiniPrep kit (Zymo Research Corp., Irvine, CA, USA) in a laminar UV flow hood in sterile conditions. Then, the DNA was placed on a 1.2% agarose gel at 80 V for 45 min in a BIORAD electrophoresis chamber (Bio-Rad Laboratories, Inc., Hercules, CA, USA). The DNA visualization was carried out in a GelMax™ photo documenter (UVP LLC, Upland, CA, USA). The amount of DNA obtained was measured in a Qubit™ fluorometer (Invitrogen, Carlsbad, CA, USA). Then, the V3-V4 region of the 16S rRNA gene was amplified using the following primers [26]: S-D-Bact-0341-b-S-17, 5′-CCTACGGGNGGCWGCAG-3′ and S-D-Bact-0785-a-A-21, 5′-GACTACHVGGGTATCTAATCC-3′.

The PCR protocol was performed by using 12.5 μL of MyTaq™ Ready Mix 1× (Bioline, London, UK), 1 μL of each primer (10 nM), 5 μL of DNA (50 ng total), and 5.5 μL of molecular grade H_2_O. The following cycle was used: 95 °C for 3 min; 25 cycles of 95 °C for 30 s, 55 °C for 30 s, 72 °C for 30 s; and 72 °C for 5 min; Illumina (2020a) [27] in a Labnet Multigene™ Gradient PCR thermal cycler (Labnet International, Edison, NJ, USA). Then, 1 μL of each PCR product was placed in an Agilent Bioanalyzer DNA 1000 chip (Agilent Technologies, Santa Clara, CA, USA) to verify the amplicon size (550 bp). The amplicons were purified with Agentcourt^®^ AMPure^®^ XP 0.8% beads (Beckman Coulter Inc., Brea, CA, USA). Thereafter, the Nextera XT Index Kit™ was used to create the library, following the Illumina (2020b) [28] protocol using 25 μL of MyTaq™ Ready Mix 1× (Bioline^®^), 5 μL of each primer (N7xx and S5xx), 5 μL of DNA, and 10 μL of molecular grade H_2_O. Each pool considered the following cycle: 95 °C for 3 min; 10 cycles of 95 °C for 30 s, 55 °C for 30 s, 72 °C for 30 s; and 72 °C for 5 min. The libraries were purified with Agencourt^®^ AMPure^®^ XP 1.2% beads. Then, 1 μL of the final PCR product library was randomly selected and placed on a Bioanalyzer DNA 1000 chip to verify the size of the amplicon expecting a size of 630 bp. Thereafter, the amplicon was quantified, normalized, (equimolarity), and sequenced with next generation massive sequencing (MiSeq; Illumina, San Diego, CA, USA) of 2 × 250 paired final readings following the 16S metagenomic protocol [27]. 

### 2.5. Bioinformatics Analyses

The DNA sequences were analyzed on MGLinux, in a VM Oracle VirtualBox using Quantitative Insights into Microbial Ecology bioinformatics software (QIIME, Boulder, CO, USA) [29]. Both forward and reverse sequences were assembled using the PEAR program [30] with an overlap of 50 bp, a minimum reading length of 430 bp, and a maximum of 470 bp, a quality criterion Q30 (one false base for every 1000 bases) with *p* < 0.0001. Then the files were converted to the FASTA format, and chimeric sequences were discarded with USEARCH [31]. Thereafter, the operational taxonomic units (OTUs) were selected with the UCLUST method [31] at 97% similarity; a representative sequence for each OTU was obtained, and the taxonomy was assigned and taken as reference to the EzBioCloud database [32]. Next, the OTUs table was built in the Biom format (biological observation matrix) [33] separating domains. A simple random rarefaction process was performed [34] in order to obtain a standardized Biom file for all pools. The Shannon and Simpson alpha diversity indexes were calculated using the standardized Biom file; the mean ± standard deviation for each index was obtained. The relative abundance for the phylum level was represented as a bar chart using Excel, and family and genus levels were visualized as heatmaps using Morpheus software (Broad Institute, Cambridge, MA, USA) [35]; hierarchical clustering (average linkage method with Euclidean distance) was used to visualize pools dendrograms. Each genus and/or species of bacteria registered for *O. turicata* was consulted in the available literature to indicate its possible pathogenic potential for tortoises or zoonotic potential for humans. 

## 3. Results 

The average number of sequences assembled was 196,564. After taxonomic designation an average of 190,769 bacterial sequences was obtained. The average number of OTUs was 44,369 (Table 1). Simple random rarefaction was made at 100,000 sequences, since at this point the number of taxa of the three pools reached asymptotes. From the standardized Biom file, the OTUs resulted in 28 phyla, 84 classes, 165 orders, 342 families, 1013 genera, and 1326 species.

**Figure 4:**
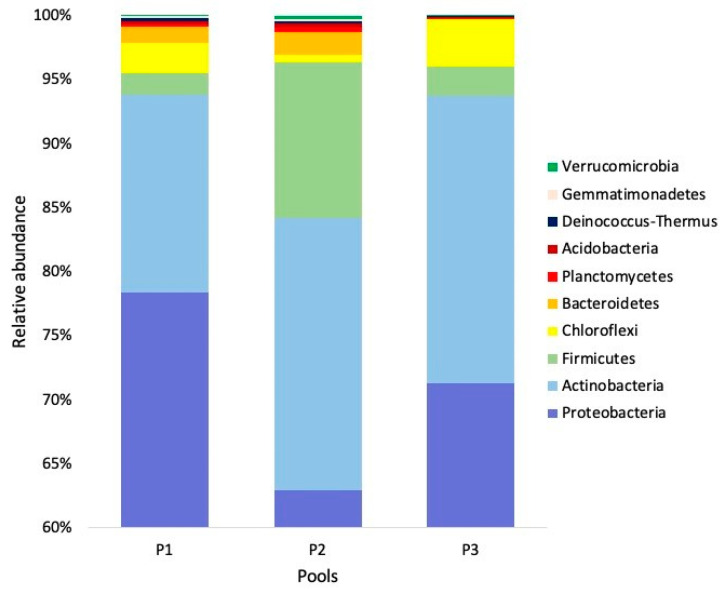
Relative abundance (%) of bacterial taxa at the phylum level from three pools of the soft tick *Ornithodoros turicata* as an ectoparasite of the Bolson tortoise (*Gopherus flavomarginatus*) in the Mapimi Biosphere Reserve, Mexico. Only the 10 most abundant phyla are shown.

While the Shannon diversity index was 8.12 ± 0.39 (SD), the Simpson diversity index was 0.86 ± 0.05. From the 28 phyla obtained, 27 (96.4%) were taxonomically classified, the rest were classified as “other”. The most abundant phyla were Proteobacteria (mean = 70.75%), Actinobacteria (mean = 19.67%), and Firmicutes (mean = 5.35 %; Figure 4). 

At the class level, 84 taxa were obtained of which 82 (97.6%) were taxonomically classified, the rest was classified as “other”. The most abundant classes were Alphaproteobacteria (mean = 56.91%), Actinobacteria (mean = 18.55%), and Gammaproteobacteria (13.65%; Table S1). From the 165 orders obtained, 158 (95.7%) were taxonomically classified, the rest were classified as “other”. The most abundant orders were Rickettsiales (mean = 55.40%), Legionellales (mean = 13.30%), Micrococcales (mean = 5.99%), Propionibacteriales (mean = 5.32%), and Srteptomycetales (mean = 5.11%; Table S1). 

**Figure 5:**
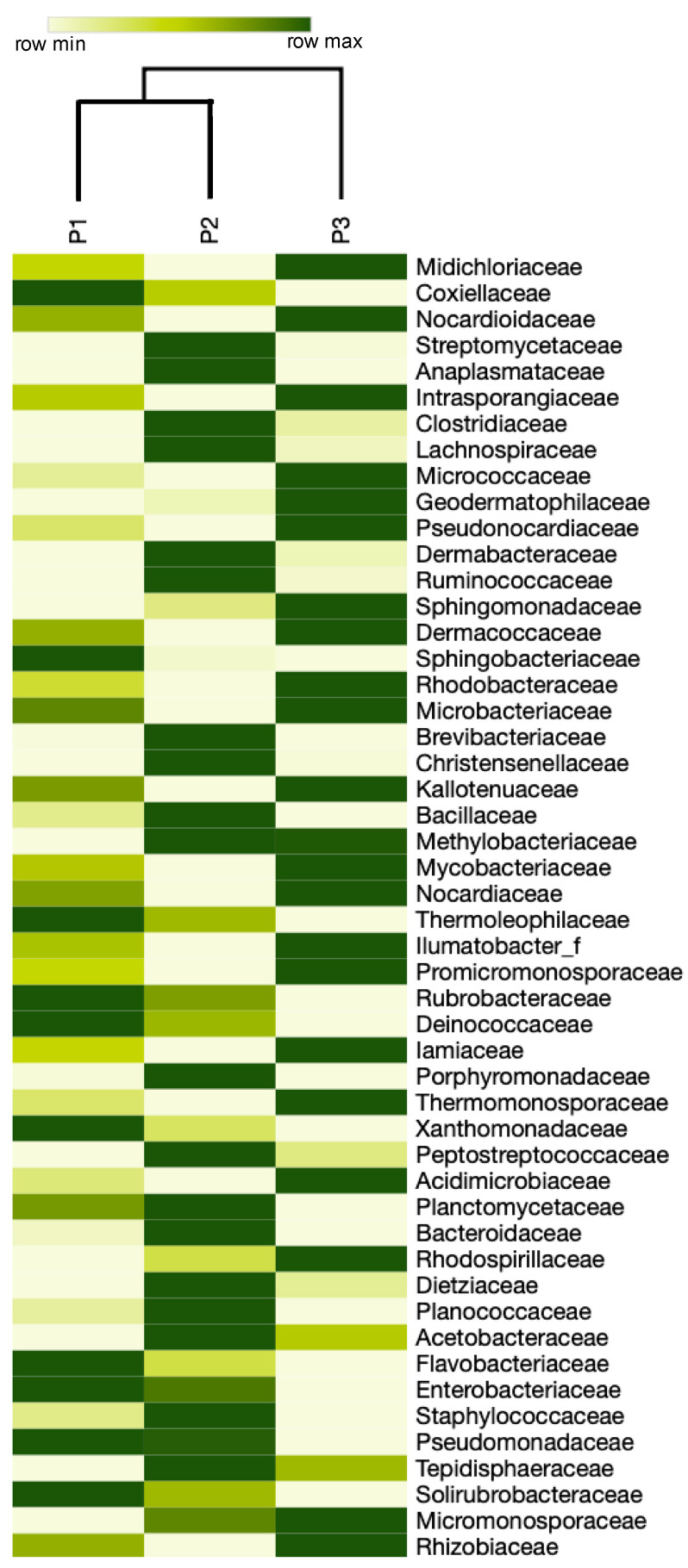
Heatmap of bacterial taxa at the family level from three pools of the soft tick *Ornithodoros turicata* as an ectoparasite of the Bolson tortoise (*Gopherus flavomarginatus*) in the Mapimi Biosphere Reserve, Mexico. Only the first 50 more abundant families having a taxonomical name are shown.

**Figure 6:**
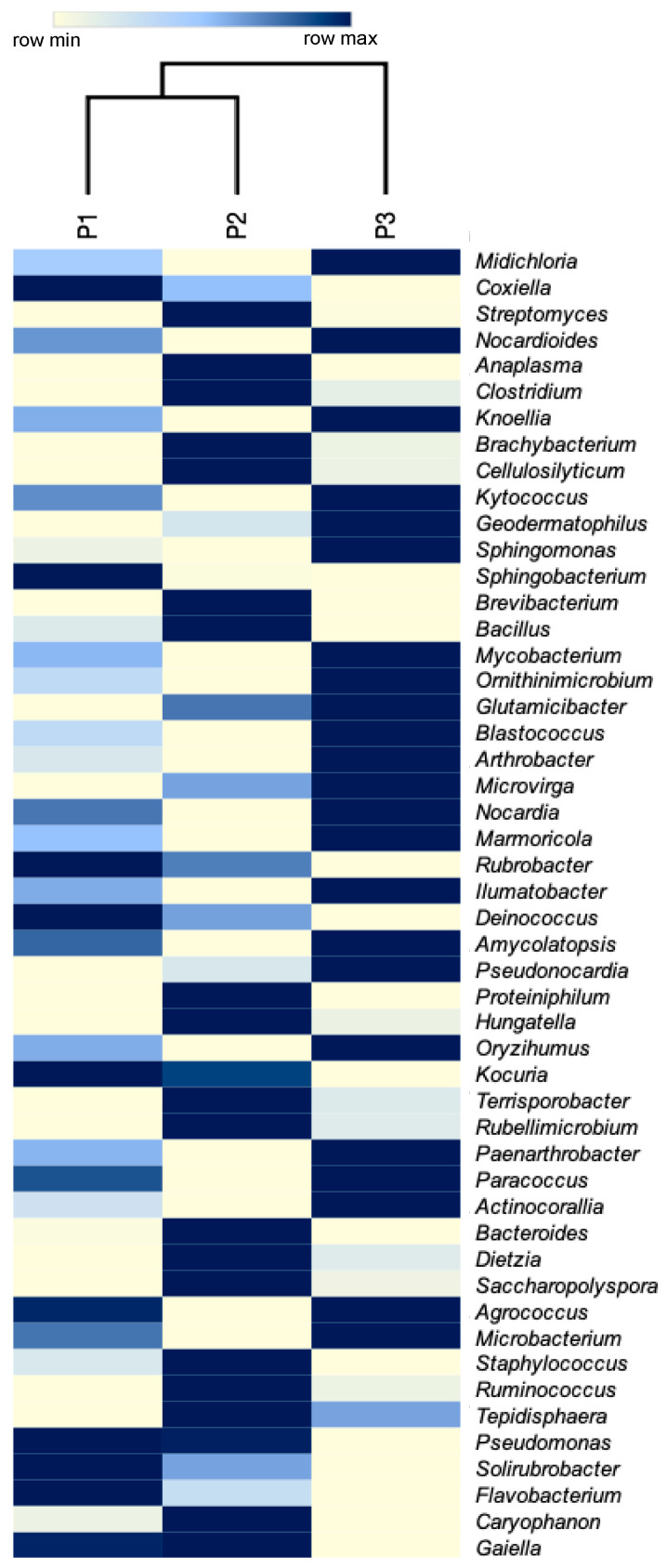
Heatmap of bacterial taxa at the genus level from three pools of the soft tick *Ornithodoros turicata* as an ectoparasite of the Bolson tortoise (*Gopherus flavomarginatus*) in the Mapimi Biosphere Reserve, Mexico. Only the first 50 more abundant genera having a taxonomical name are shown.

At the family level, 342 taxa were obtained of which 325 (95.0%) were taxonomically classified, the rest were classified as “other”. The most abundant families were Midichloriaceae (mean = 51.13%), Coxiellaceae (mean = 13.26%), Nocardioidaceae (mean = 5.29%), Streptomycetaceae (mean = 5.10%), and Anaplasmataceae (mean = 4.29%; Figure 5). From the 1013 genera obtained, 897 (88.54%) were taxonomically classified, the rest were classified as “other”. The most abundant genera were *Midichloria* (mean = 51.13%), *Coxiella* (mean = 13.25%), *Streptomyces* (5.08%), *Nocardioides* (mean = 5.06%), *Anaplasma* (mean = 4.29%), and *Clostridium* (mean = 2.19%; Figure 6; Table S1). The genus *Mycoplasma*, a potential pathogen of the *Gopherus* spp., was registered in low abundance (mean = 3.33 × 10^−6^ %). Additionally, 1326 species were obtained, 918 (69.2%) were classified as “other”, 268 (20.3%) have a taxonomical key, and 140 (10.5%) have a taxonomical name (Table 2). The most abundant species were *Midichloria*-like bacteria (mean = 50%), *Coxiella*-like bacteria (mean = 13%), *Streptomyces* sp. (mean = 5%), and *Nocardioides* sp. (mean = 5%). However, considering only bacteria with a complete taxonomical name the most abundant was *Midichloria mitochondrii* (mean = 0.72%; Table S1). *Pasteurella testudinis*, a potential pathogen of the *Gopherus* spp., was registered in low abundance (mean = 1.33 × 10^−5^%). The bovine and ovine tick-borne bacteria *A. marginale* and *A. ovis*, respectively, were recorded in low abundances; also, the zoonotic bacteria *Coxiella burnetii, Anaplasma phagocytophilum,* and *Neoehrlichia* sp. were part of the *O. turicata* microbiota (see relative abundances in Table S1). 

## 4. Discussion

Our working hypothesis stated that the microbiota of *O. turicata* would contain potentially pathogenic bacteria for the Bolson tortoise *Gopherus flavomarginatus*, other animals, and humans. Therefore, and based on the obtained results, this study demonstrated the presence of some potentially pathogenic and zoonotic bacteria in the microbiota of *O. turicata*.

The most abundant phyla were Proteobacteria, Actinobacteria, and Firmicutes. This is similar to that reported for *Ambylomma tuberculatum* ticks infesting the Gopher tortoise (*G. polyphemus*) in Mississippi, USA, with 89%, 9%, and 1% of the relative abundance, respectively [36]. Currently, Proteobacteria is the broadest phylum in the Bacteria domain [37]. Some of the tick species that have shown dominance of this phylum in their microbiota are *Argas japonicus* in China (42–97%) [38], *Hyalomma dromedarii* in Saudi Arabia (98%) [39], *Dermacentor marginatus* and *D. reticulatus* in Slovakia (60%) [40], *D. marginatus* (97%), *Haemaphysalis punctata* (98%), *Ixodes ricinus* (88%), and *Rhipicephalus sanguineus sensu lato* (99%) in Spain [41], and *I. scapularis* in Massachusetts and Texas, USA (73–100%; [8]).

Alphaproteobacteria and Gammaproteobacteria classes were the most abundant in *O. turicata* microbiota, as has been observed in other tick species [41]. These classes are relevant in the microbiota of all tick species because they group the main intracellular endosymbionts (*Arsenophonus*-like, *Coxiella*-like, *Fransicella*-like, *Midichloria*-like, *Rickettsia*-like, and *Wolbachia*-like) that inhabit the organs of these arthropods [6,42]. These endosymbionts have generally been reported to predominate in the arthropod microbiota and may interfere with the transmission dynamics of pathogenic bacteria [43,44]. In the present study, *O. turicata* showed only two genera of intracellular endosymbionts, *Midichloria* and *Coxiella*. An unidentified species *Midichloria*-like endosymbiont was the dominant taxon in the *O. turicata* microbiota, although *Candidatus Midichloria mitochondrii* (CMM) species was also abundant in this tick. CMM has been reported in a large number of tick genera (*Amblyomma* spp., *Dermacentor* spp., *Haemaphysalis* spp., *Hyalomma* spp., *Ixodes* spp., and *Rhipicephalus* spp.) in different countries of Europe, Asia, and North America [41,45,46]. It is considered a facultative non-pathogenic mutualistic bacterium that lodges in the reproductive tissues of female ticks, specifically in the ovarian mitochondria, where they sometimes invade and destroy this organ [47,48]. By remaining in this organ, vertical transmission to the next generation is carried out. However, its presence has also been recorded in salivary glands from which it is transmitted to vertebrate hosts during tick feeding [49]. Since all the body of *O. turicata* was analyzed in the present study, it is not possible at this time to determine in which organs occur both unknown *Midichloria*-like and CMM. However, it is feasible that they occur in the reproductive organs since some studies have analyzed the possible evolutionary process of intracellular endosymbionts of arthropods, and apparently transovarian transmission is the common factor for all species [50]. Bacterial diversity studies in each organ of *O. turicata* are required to clarify this question.

The second most abundant bacterial species in *O. turicata* microbiota in our study was a *Coxiella*-like endosymbiont. This type of endosymbiont does not appear to be pathogenic and are relatively common in the microbiota of various tick species around the world [51,52]. These microorganisms are known to infect tick ovaries and then transmission occurs vertically through the egg cytoplasm, but they have also been found in Malpighi tubules where they may provide essential nutrients to their host [7]. It should be noted the presence of *C. burnetii* in the present study. This is a zoonotic bacterium that causes Q-fever in both animals and humans. In particular, for the *Ornithodoros* genus there are reports of *C. burnetii* in *O. tartakovskyi*, *O. papillipes* and *O. alactagalis* in the former Soviet Union [53], *O. moubata* in Japan [54], and *O. sonrai* in Senegal [55]. According to Balashov and Daiter (1973) [53] and Eldin et al. (2017) [56], transmission of *C. burnetii* is transovarian in most ticks, except for *Ixodes holocyclus*, *O. hermsi*, and *O. turicata* in which it is transestadial. Direct transmission of *C. burnetii* from infected ticks to humans is not well documented and may occur only rarely in nature [57]. However, the main transmission route is via inhalation of contaminated fecal material from ticks [58]. Since the personnel that monitor *G. flavomarginatus* populations each year in the Mapimi Biosphere Reserve should manipulate tortoises, it is advisable to take precautions to avoid this airborne infection.

*Mycoplasma* spp. and *Pasteurella* spp. are bacterial genera that have been reported as part of the microbiota of hard ticks, as in *Ixodes simplex* and *I. ventalloi,* respectively [59,60]. In the present study with *O. turicata*, the presence of *Mycoplasma* spp. and *Pasteurella testudinis* in low abundances was recorded. An interesting fact is that these bacteria along with some viruses have been reported as some of the possible causes of upper respiratory disease (URTD) in tortoises, causing a decrease in the populations of *G. agassizii* and *G. polyphemus* in the USA [61,62]. It is still unknown if these bacteria can be transmitted directly to the host, and in the case of *G. flavomarginatus* if they could cause disease. For now, these bacteria should remain as potential pathogens for this tortoise, until further studies of vector competence discard this possibility.

Other relevant species recorded in *O. turicata* microbiota were the obligate intracellular bacteria *Anaplasma phagocytophilum*, *A. marginale*, *A. ovis*, and *Neoehrlichia* sp. These species are important for animal and human health because they typically infect hematopoietic or endothelial cells [63]. *Anaplasma phagocytophilum* is a tick-borne pathogen that causes human granulocytic anaplasmosis mainly in Asia, Europe, and the USA; *A. marginale* and *A. ovis* are worldwide tick-borne bacteria causing bovine and ovine anaplasmosis respectively [64,65]. The genus *Neoehrlichia* was discovered in 1999 as an *Ehrlichia*-like bacterium [66], which was later described as *Candidatus Neoehrlichia mikurensis* [67], an emerging tick-borne pathogen detected in ticks and rodents, causing human systemic inflammatory syndrome in Asia and Europe [68,69]. In America, *Candidatus Neoehrlichia lotoris* was registered in a free-living raccoon associated with tick-infested populations [70], which suggests that it is transmitted to mammals by ticks. Undoubtedly, specific molecular studies are needed to determine which *Neoehrlichia* species is the one registered in *O. turicata*, nevertheless, its zoonotic potential is latent.

According to Gofton et al. (2015b) [71], a limitation of 16S bacterial community profiling in ticks is that a high proportion of sequences will belong to bacterial endosymbionts, as CMM as observed in *O. turicata* in the present study. This high abundance may mask the presence of less abundant bacteria with zoonotic potential, as for example the genus *Borrelia*, which was not detected in our study. Species such as *B. mazzottii* recorded in *O. talaje* in Mexico [72], as well as *B. parkeri* in *O. parkeri*, *B. hermsii* in *O. hermsi,* and *B. turicatae* in *O. turicata* in the USA [73], are important indicators of the presence of this bacterial genus in the *Ornithodoros* ticks of America. Perhaps, by carrying out detailed molecular studies in *O. turicata* in the Mapimi Biosphere Reserve, *Borrelia* and other zoonotic bacteria such as *Rickettsia* could be identified. In the same way, since this massive sequencing technique does not reach the species level in many genera documented here for *O. turicata*, it is necessary to carry out specific PCR studies for the most relevant bacteria involved in the biology of this tick and those bacteria important for the health of animals and humans.

It is important to comment that the present study was carried out at a single time of the year, without distinguishing sexes of *O. turicata* to provide an initial overview of the microbiota of this tick species. However, various studies indicate that the microbiota of ticks is dynamic and can change among seasons of the year due to the effect of temperature and humidity, between males and females, between different feeding statuses, among life stages, etc. [3,4]. Therefore, it is guaranteed that detailed studies on these issues will further be carried out.

Finally, the high number of bacterial taxa recorded for *O. turicata* in the present study could be due to the variety of hosts that this tick parasitizes within the burrows of *G. flavomarginatus*, performing a rapid feeding process that usually lasts an hour on average [14,74]. In this way the general feeding habits of *O. turicata* may be keeping pathogenic microbes in circulation, thereby ensuring their survival in the ecosystem. In our study, all *G. flavomarginatus* tortoises found carrying *O. turicata* ticks were apparently healthy, having the possible ecological role of reservoirs of pathogenic bacteria [75,76]. The fact that tortoises have long lives can favor the maintenance of pathogen cycles under normal conditions [77,78]. However, after thousands of years of coevolution, a microbial balance between *O. turicata* and *G. flavomarginatus* must have been reached, although it is not yet known whether this tortoise’s defense mechanism is resistance (i.e., capacity to limit pathogen loads) or tolerance (i.e., capacity to survive damage caused by a given pathogen load) [79,80,81]. To clarify this enquiry, it will be necessary to determine the bacteria that *G. flavomarginatus* carries in the blood and whether they were transmitted by *O. turicata*. Studies of resistance mechanisms and vector competence in this tortoise will also be relevant from the point of view of immunity and the eco-epidemiology of zoonotic diseases, respectively.

## 5. Conclusions

Our study established the general relative abundance of bacterial taxa in the soft tick *O. turicata*, documenting the presence of potentially pathogenic species for the Bolson tortoise *G. flavomarginatus*, other animals and humans in the Mapimi Biosphere Reserve, Durango, Mexico. The most abundant phyla were Proteobacteria, Actinobacteria, and Firmicutes. Additionally, Alphaproteobacteria and Gammaproteobacteria classes were the most abundant in *O. turicata* microbiota. The most abundant species were *Midichloria*-like and *Coxiella*-like (endosymbionts of ticks). *Mycoplasma* spp. and *Pasteurella testudinis*, both potentially pathogenic to the Bolson tortoise, were registered. Additionally, the bovine and ovine tick-borne pathogens *A. marginale* and *A. ovis*, respectively, as well as the zoonotic bacteria *A. phagocytophilum*, *Coxiella burnetii*, and *Neoehrlichia* sp. were founded in *O. turicata*. Future studies on soft ticks should expand our knowledge about what components of the microbiota are notable across multiple host–microbe dynamics. Likewise, studies are required to better understand the reservoir competence of this tortoise, considered the largest terrestrial reptile in North America distributed throughout the Chihuahuan Desert since the late Pleistocene.

## Figures and Tables

**Table 1:** 16S rRNA V3-V4 region sequences obtained from soft tick *Ornithodoros turicata* pools as an ectoparasite of the Bolson tortoise (*Gopherus flavomarginatus*) in the Mapimi Biosphere Reserve, Mexico.

Pool	Total	Assembled	Discarded	ChS	QS	BS	OTUs
P1	424,827	278,006	146,821	1580	276,426	272,339	56,998
P2	457,353	202,476	254,877	1633	200,843	195,622	41,791
P3	159,929	109,211	50,718	278	108,616	104,347	34,318
Mean	347,370	196,564	150,805	1164	195,295	190,769	44,369

Abbreviations are ChS = chimeric sequences eliminated, QS = quality sequences after chimeras elimination, BS = bacteria sequences after taxonomy designation, and OTUs = operational taxonomic units.

**Table 2:** Bacterial species found in the soft tick *Ornithodoros turicata* as an ectoparasite of the Bolson tortoise (*Gopherus flavomarginatus*) in the Mapimi Biosphere Reserve, Mexico ^1^.

Bacterial Species	Bacterial Species	Bacterial Species
*Actinocorallia libanotica*	*Kribbia dieselivorans*	*Phycicoccus jejuensis*
*Actinophytocola timorensis*	*Kytococcus aerolatus*	*Phytomonospora endophytica*
*Aeromicrobium panaciterrae*	*Kytococcus schroeteri*	*Piscicoccus intestinalis*
*Agrococcus terreus*	*Kytococcus sedentarius*	*Planosporangium thailandense*
*Anaerocella delicata*	*Lactobacillus sakei*	*Propionibacterium acnés*
*Anaplasma marginale*	*Lactococcus lactis*	*Rubellimicrobium mesophilum*
*Anaplasma ovis*	*Lentzea kentuckyensis*	*Saccharopolyspora hirsuta*
*Anaplasma phagocytophilum*	*Leucobacter celer*	*Salmonella entérica*
*Anseongella ginsenosidimutans*	*Leucobacter chromiireducens*	*Segetibacter koreensis*
*Arthrobacter halodurans*	*Leucobacter tardus*	*Seinonella peptonophila*
*Bacillus cecembensis*	*Leucobacter zeae*	*Serinicoccus profundi*
*Bacillus halosaccharovorans*	*Lysobacter dokdonensis*	*Sinomonas mesophila*
*Bacillus niacini*	*Magnospira bakii*	*Skermanella aerolata*
*Bauldia litoralis*	*Marmoricola bigeumensis*	*Skermanella stibiiresistens*
*Blautia wexlerae*	*Marmoricola pocheonensis*	*Smaragdicoccus niigatensis*
*Brachybacterium ginsengisoli*	*Marmoricola terrae*	*Sphingoaurantiacus polygranulatus*
*Brachybacterium phenoliresistens*	*Massilia brevitalea*	*Sphingobacterium changzhouense*
*Brachybacterium squillarum*	*Methylobacterium oxalidis*	*Sphingobacterium hotanense*
*Brachybacterium zhongshanense*	*Micromonospora taraxaci*	*Sphingobacterium mucilaginosum*
*Cellulosilyticum lentocellum*	*Midichloria mitochondrii*	*Sphingobacterium siyangense*
*Citricoccus yambaruensis*	*Mobilicoccus pelagius*	*Sphingobacterium suaedae*
*Clavibacter michiganensis*	*Mycobacterium rutilum*	*Stackebrandtia cavernae*
*Clostridium butyricum*	*Nocardia brevicatena*	*Staphylococcus cohnii*
*Clostridium chartatabidum*	*Nocardia puris*	*Staphylococcus hominis*
*Clostridium máximum*	*Nocardioides aestuarii*	*Staphylococcus saprophyticus*
*Coxiella burnetii*	*Nocardioides agariphilus*	*Staphylococcus sciuri*
*Cronobacter dublinensis*	*Nocardioides albertanoniae*	*Staphylococcus succinus*
*Dermabacter vaginalis*	*Nocardioides daedukensis*	*Stenotrophobacter namibiensis*
*Eubacterium eligens*	*Nocardioides glacieisoli*	*Streptomyces bacillaris*
*Flavobacterium anatoliense*	*Nocardioides islandensis*	*Streptomyces baliensis*
*Geodermatophilus arenarius*	*Nocardioides kongjuensis*	*Streptomyces barkulensis*
*Geodermatophilus nigrescens*	*Nocardioides lianchengensis*	*Streptomyces cacaoi*
*Geodermatophilus obscurus*	*Nocardioides luteus*	*Streptomyces drozdowiczii*
*Geodermatophilus saharensis*	*Nocardioides mesophilus*	*Streptomyces fenghuangensis*
*Geodermatophilus soli*	*Nocardioides pyridinolyticus*	*Streptomyces glaucosporus*
*Glutamicibacter creatinolyticus*	*Nocardioides tritolerans*	*Streptomyces mangrovi*
*Glutamicibacter nicotianae*	*Ornithinimicrobium humiphilum*	*Streptomyces murinus*
*Helcobacillus massiliensis*	*Ornithinimicrobium kibberense*	*Streptomyces thermoviolaceus*
*Janibacter alkaliphilus*	*Ornithinimicrobium murale*	*Syntrophomonas curvata*
*Janibacter corallicola*	*Ornithinimicrobium pekingense*	*Syntrophomonas wolfei*
*Kallotenue papyrolyticum*	*Ornithinimicrobium tianjinense*	*Taibaiella chishuiensis*
*Kibdelosporangium aridum*	*Paenarthrobacter nitroguajacolicus*	*Terracoccus luteus*
*Kineococcus radiotolerans*	*Paraburkholderia caledonica*	*Tetrasphaera elongata*
*Kineosphaera limosa*	*Paracoccus saliphilus*	*Tetrasphaera remsis*
*Knoellia aerolata*	*Paracoccus sphaerophysae*	*Tetrasphaera vanveenii*
*Knoellia sinensis*	*Pasteurella testudinis*	*Thermobaculum terrenum*
*Kocuria flava*	*Phycicoccus endophyticus*	

^1^ Relative abundance is presented in Table S1

## Supplementary Materials

The following are available online at https://www.mdpi.com/2079-7737/9/9/275/s1, Table S1: Relative abundance of bacterial taxa from three pools of the soft tick *Ornithodoros turicata* as an ectoparasite of the Bolson tortoise (*Gopherus flavomarginatus*) in the Mapimi Biosphere Reserve, Mexico, Supplementary files (P1.fasta, P2.fasta and P3.fasta). The files used in this study were also deposited into the NCBI Sequence Read Archive (SRA) database (SRA Accession Number: PRJNA649587).
